# Design and Optimisation of Elliptical-Shaped Planar Hall Sensor for Biomedical Applications

**DOI:** 10.3390/bios12020108

**Published:** 2022-02-10

**Authors:** Shah Mukim Uddin, Abkar Sayad, Jianxiong Chan, Efstratios Skafidas, Patrick Kwan

**Affiliations:** 1 Department of Medicine, Royal Melbourne Hospital, The University of Melbourne, Melbourne, VIC 3050, Australia; suddin1@student.unimelb.edu.au (S.M.U.); jianxiong.chan@monash.edu (J.C.); sskaf@unimelb.edu.au (E.S.); 2 Department of Neuroscience, The Alfred Centre, Central Clinical School, Monash University, Melbourne, VIC 3004, Australia; abkar.sayad@monash.edu; 3 Department of Electrical and Electronic Engineering, Melbourne School of Engineering, The University of Melbourne, Melbourne, VIC 3010, Australia

**Keywords:** magnetometer, planar hall sensor, magnetic bead, microfabrication, biosensor, magnetic immunoassay

## Abstract

The magnetic beads detection-based immunoassay, also called magneto-immunoassay, has potential applications in point-of-care testing (POCT) due to its unique advantage of minimal background interference from the biological sample and associated reagents. While magnetic field detection technologies are well established for numerous applications in the military, as well as in geology, archaeology, mining, spacecraft, and mobile phones, adaptation into magneto-immunoassay is yet to be explored. The magnetic field biosensors under development tend to be multilayered and require an expensive fabrication process. A low-cost and affordable biosensing platform is required for an effective point-of-care diagnosis in a resource-limited environment. Therefore, we evaluated a single-layered magnetic biosensor in this study to overcome this limitation. The shape-induced magnetic anisotropy-based planar hall effect sensor was recently developed to detect a low-level magnetic field, but was not explored for medical application. In this study, the elliptical-shaped planar hall effect (EPHE) sensor was designed, fabricated, characterized, and optimized for the magneto-immunoassay, specifically. Nine sensor variants were designed and fabricated. A customized measurement setup incorporating a lock-in amplifier was used to quantify 4.5 µm magnetic beads in a droplet. The result indicated that the single-domain behaviour of the magnetic film and larger sensing area with a thinner magnetic film had the highest sensitivity. The developed sensor was tested with a range of magnetic bead concentrations, demonstrating a limit of detection of 200 beads/μL. The sensor performance encourages employing magneto-immunoassay towards developing a low-cost POCT device in the future.

## 1. Introduction

The limitations of solid-phase binding-based biosensors include the uneven distribution of tagged particles, inability to measure the three-dimensional region, reduced sensitivity due to background interference from the biological sample, nonlinear characteristics, non-specific detection, complexity on integration, and performance limitation due to detection circuitry. Magnetic biosensing may overcome these limitations [1,2,3]. The advantages of magnetic biosensing include minimized background signals from the biological sample, three-dimensional detection ability, reduced matrix effect, chemical stability, quantification, biocompatibility, and larger active surface area for immobilization. In addition, magnetic beads can increase signal transduction and amplify target recognition, improving biosensors’ sensitivity. For decades, magnetic field sensing technology has been utilized in data storage devices and industrial sensor applications because of its reliability, versatility, and ruggedness. These applications are the foundation of the magnetic detection techniques developed for biosensing. The magnetic bead-tagged analytes’ presence can be detected via the change of a magnetic field from the magnetic beads when an external magnetic field is induced [4]. Several methods have been developed for sensing the magnetic beads that have been reviewed by multiple research groups [2,5,6,7]. The magnetic detection methods are mainly classified based on the physical parameter measured, such as magnetic permeability, magnetic remanence, and magnetoresistance. Detection of magnetic bead density rather than single bead detection is required for magnetic immunoassay application, due to the target molecule’s binding challenge on a smaller sensing area [2]. Hence, the sensitivity is expressed as the “detectable beads per area” for solid-phase assays [8].

Magnetoresistive sensors have tremendous potential for magneto-immunoassay application due to the miniature size, low noise, and low fabrication cost compared with other magnetometers. Magnetoresistive sensors are constructed as single or multilayer magnetic films to detect the stray magnetic field [9,10,11]. Magnetoresistive sensors are widely used in information storage technology, automotive, and telecommunication industries. In addition, several research groups have exploited the applications of magnetoresistive sensors for biomolecular recognition [12,13,14]. There are three major categories based on the structures and working principles, such as anisotropic magnetoresistance sensors (AMR), giant magnetoimpedance sensors (GMR) [15,16], and tunnelling magnetoresistance sensors (TMR). The magnetoresistive sensor has good linearity, a simplified fabrication process, and shape flexibility. The magnetoresistive sensor’s magnetization aligns with the magnetic field and changes the electrical resistivities under an external magnetic field based on the ferromagnetic material phenomenon. The variation of the longitudinal resistivity due to the external magnetic field is called the anisotropic magnetoresistive (MR) effect, and the variation of the transversal resistivity is called the planar hall effect (PHE) [17]. The electrical resistance depends on the relative angle between the applied current’s direction and the orientation of the magnetization [18]. The ferromagnetic materials have low resistance when their directions are perpendicular to the current, and high resistance when parallel.

The PHE sensor’s signal depends on the angle between the current’s direction and the magnetization in the magnetic thin film. The advantages are linearity in low magnetic field [19], high signal-to-noise ratio [20,21,22], and low thermal drift [23] compared with AMR, which makes it promising for magneto-immunoassay application. Tamanaha et al. [2] have reported a comprehensive comparison of the magnetic label per area, suggesting that hall sensors achieved higher sensitivity within a relatively small detection area than other methods. The magnetic material’s requirements are uniform magnetization and the reversible magnetic direction without hysteresis. The thin film needs magnetic anisotropy to achieve this characteristic, whereas the easy axis is parallel to the current direction. Different techniques to achieve the magnetic anisotropy were reviewed by Mor et al. [17], including field-induced magnetic anisotropy [24,25,26,27], spin valve structure [28,29,30], bridge structure [24,25,26,31,32,33,34], and shape-induced magnetic anisotropy [22,35,36,37]. The shape-induced approach has the unique advantage of simplified fabrication with a single ferromagnetic layer structure. Mor et al. [36] have reported that the elliptical shape exhibits better performance than the rectangle shape to achieve the effective single-domain behaviour. The single-domain characteristic is noticeable from the ellipse’s axis ratio of 6:1 and improves with increasing axis ratio.

The elliptical-shaped planar hall (EPHE) sensors induce uniaxial magnetic anisotropy parallel to the ellipse’s long axis. A constant current is driven along the ellipse’s long axis, and the transverse voltage is measured across the ellipse’s short axis to detect the magnetic field. The EPHE sensor is attractive compared with other magnetoresistive sensors due to its low-temperature dependence [38], simple fabrication, and high resolution [17,21,22,37]. Grosz et al. reported PHE sensors with an equivalent magnetic noise or resolution of ~200 pT/√Hz [21], and the noise can be reduced to ~5 pT/√Hz by adding a magnetic flux concentrator [11,37,39]. The increased sensitivity of the magnetometer also reduces the dynamic range [38].

Given its unique properties, EPHE has considerable potential for point-of-care medical applications, due to its straightforward and less expensive fabrication requirement than other magnetoresistive sensors. However, the capabilities of the EPHE sensor for medical application have not been explored. Careful optimization of the sensitivity and the dynamic range would be required for biomarker-specific medical applications. The ability to adjust sensitivity and dynamic range is desirable in medical diagnostics, as it would allow tailored design for a wide variety of analyte quantification. In this study, we designed and fabricated a range of EPHE sensors with different sensing areas and thicknesses to determine the optimal dimensional configuration for magnetic bead quantification.

## 2. Materials and Methods

### 2.1. Physical Background

The magnetic moment has no preferential direction in the absence of an external magnetic field for the isotropic magnetic material. In contrast, the magnetic moment aligns with the energetically favourable direction (named ‘easy axes’) of spontaneous magnetization for the magnetic anisotropic material. The two opposite directions along the easy axis are equivalent, and the magnetization direction can be either. The uniaxial magnetic anisotropy is parallel to the ellipse’s long axis determined by the geometry. To quantitate the magnetic field, a current is driven along the ellipse’s long axis, and the transverse voltage is measured across the ellipse’s short axis, which the PHE generates. The PHE is expressed by the equation [40]:

(1) $$V_{o}=j\left(\frac{\rho_{∥}-\rho_{⊥}}{2}\right)\sin2\theta$$

Here, θ is the angle between the magnetization (M) and current density (j). $\rho_{∥}$ and $\rho_{⊥}$ are the parallel and perpendicular resistivities to the current, respectively. $V_{o}$ is the hall voltage which is the perpendicular electric field to the current density. The magnitude of the hall voltage is reversely proportional to the film’s thickness given by the equation below [41]:

(2) $$V_{o}=\frac{\mathrm{jH}}{t}R_{H}$$

Here, H is the magnetic field strength induced by the magnetic beads, t is the film’s thickness, and $R_{H}$ is the hall coefficient. When H is small compared with the effective anisotropy field ($H_{\mathrm{eff}}$), the sensitivity is [22]:

(3) $$S=\frac{V_{o}}{H}=j\left(\frac{\rho_{∥}-\rho_{⊥}}{t}\right)\frac{1}{H_{\mathrm{eff}}}$$

$H_{\mathrm{eff}}$ is the sum of the shape-induced anisotropy ($H_{k}$) and the growth-induced anisotropy ($H_{a}$). In this study, the magnetic field was not magnetically induced during the thin film deposition, hence $H_{a}=0$. So, the sensitivity is:

(4) $$S=\frac{j.\Delta \rho }{t.H_{k}}$$

Here, Δρ is the AMR amplitude. $H_{\mathrm{eff}}$ decreases with the decreasing film thickness [38]; hence, increasing the sensitivity. The sensor has two noise components, i.e., pink noise and thermal noise. The pink noise is negligible at the sufficiently high frequency. The thermal noise depends on the film’s thickness and excitation current. The noise analysis was detailed by Grosz et al. [22].

### 2.2. Design and Microfabrication

**Figure 1:**
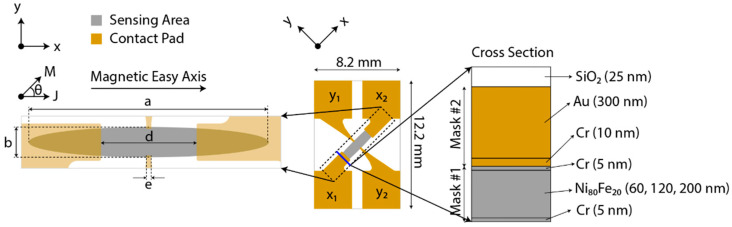
Geometry of the EPHE sensor with the layer composition. The current excitation node is x_1_ and x_2_ along the magnetic easy axis. The voltage measuring node is y_1_ and y_2_. The exposed permalloy by the gold film defines the sensing area. Cr/Ni_80_Fe_20_/Cr was fabricated with photomask #1, and Cr/Au/Cr was fabricated with photomask #2.

Figure 1 shows the schematics of the EPHE sensor’s geometry tailored for biomedical applications. Three variants were designed based on the sensing area, maintaining the axis ratio (a/b) to 8, and the corresponding dimension of each variant is detailed in Table 1. Two chromium photomasks were fabricated to fabricate 62 sensors on a 4–inch substrate. The fabrication process is detailed in Appendix A.1. In brief, the fabrication process involved electron beam-physical vapor deposition (EBPVD), photolithography, wet etching, and lift-off. Figure 1 shows the layer composition fabricated on an undoped glass wafer. The elliptical feature was patterned with photomask #1, which comprised permalloy (i.e., 60 nm, 120 nm, and 200 nm) capped with chromium (5 nm). The bottom Cr film acted as adherent to the substrate and the top as antioxidants. The contact pad was patterned with photomask #2, which comprised gold (300 nm) adhered with chromium (10 nm). The contact pad was >1.5 times thicker than the elliptical feature. Afterwards, SiO_2_ (25 nm) was deposited to passivate. The wafers were coated with photoresist before being diced into single sensors to protect them from damage. The sensors were washed before measurements with acetone, followed by isopropyl alcohol (IPA), to remove the protective photoresist.

In addition to the thicknesses mentioned in Table 1, a 300-nm thick elliptical pattern was fabricated, which was unsuccessful due to thermal expansion causing film crack. Additional information is available in Appendix A.4.

### 2.3. Experimental Setup and Measurement

**Figure 2:**
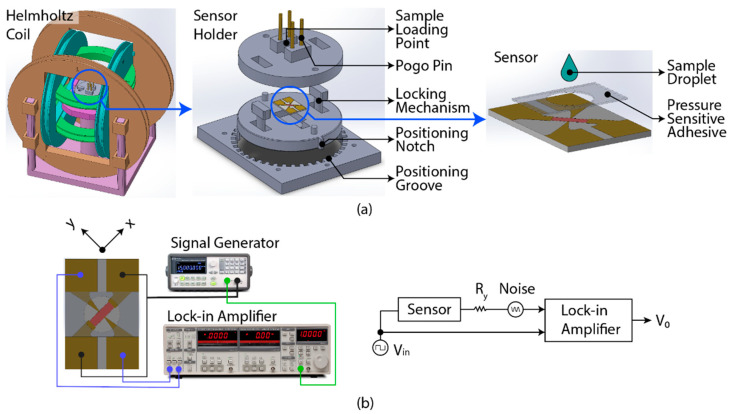
Experimental setup: (**a**) EPHE sensor mounting mechanism to the 3-axis Helmholtz coil with the sensor holder. The Helmholtz coil allows the magnetic excitation, and the sensor holder allows manual rotation of the sensor at different magnetization angles. (**b**) Sensor’s measurement setup and simplified diagram. The signal generator excites the sensor with a constant AC, and the lock-in amplifier measures the hall voltage.

The experiment setup was mainly constructed with the 3D-printed components (3D printer model: Objet Eden260VS, Eden Prairie, MN, USA and material id: RGD720). Figure 2a illustrates the sensor mounting mechanism comprising the Helmholtz coil and the sensor holder. The 3-axis Helmholtz coil was constructed to saturate the sensor’s magnetization along the easy axis and excite the magnetic beads for measurement. The sensor holder was used to place the sensor in the Helmholtz coil’s centre to establish the contact pad’s electrical connection with the measuring equipment and to allow measurement at different magnetic excitation angles. The sensor was assembled with pressure-sensitive adhesive to contain the sample droplet on the sensing area. Additional details of the sensor mounting mechanism are available in Appendix A.2 and Appendix A.3.

Figure 2b shows the measurement circuitry. First, the sensor’s magnetization was saturated with a 50 Oe magnetic field generated by the Helmholtz coil for 3 s along the elliptical’s long axis to reset the magnetization, referred to as ‘magnetic pulse’. Then, the sensor was excited with a constant alternating current (1 kHz square wave) along the elliptical’s long axis. Simultaneously, the potential difference (V_o_) was measured with the lock-in amplifier (model no. SR830, Stanford Research Systems, Sunnyvale, CA, USA) along the elliptical’s short axis, referred to as ‘hall voltage’. All sensor measurements were carried out at room temperature (~22 °C).

The hall voltage was measured at each rotating step of the sensor holder to characterize the sensor’s single-domain behaviour. The sensor holder’s position was changed manually. The sensor’s sensitivity and dynamic range was determined by applying a magnetic field (0–15 Oe) at 45° to the current direction using the Helmholtz coil. Water-diluted tosylactivated dynabeads M-450 (manufacturer: Thermo Fisher Scientific, Waltham, MA, USA, catalogue number 14,013) were utilized to determine the sensor’s detection limit and limit of linearity for magneto-immunodiagnostic application. The magnetic beads diameter was 4.5 μm. Then, 3 μL magnetic bead solution was dispensed and dried on the sensor surface to settle the beads. The sample was magnetized at 90° to the excitation current with the Helmholtz coil during measurement. The sensor was measured two times to quantitate the magnetic beads, i.e., (1) before sample loading (V_pre_), and (2) after sample loading (V_post_). To avoid unstable measurements due to the Brownian motion of the beads, there was a ~10 min waiting time between sample loading and measurement to allow the solution to evaporate and settle the beads on the sensor surface. The percentage of hall voltage change was measured based on Equation (5), which indicates the magnetic bead’s concentration.

(5) $$\%\Delta V_{0}=\frac{V_{\mathrm{post}}-V_{\mathrm{pre}}}{V_{\mathrm{pre}}}\times 100$$

The sensor’s dynamic range was determined by the linear range of the sensor response. The sensitivity (S) of the sensor’s linear region was measured with the following equation:

(6) $$S=\frac{\Delta V_{0}}{\Delta H}$$

## 3. Results

### 3.1. Magnetic Behaviour

**Figure 3:**
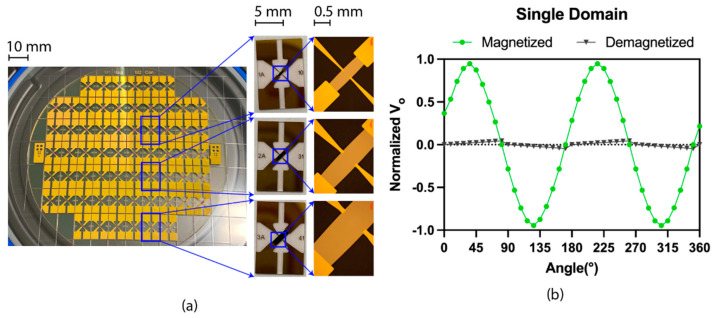
(**a**) 62 sensors in 4–inch glass wafer after dicing and three sensor variants based on the sensing area with the microscopic image; (**b**) effective single-domain behaviour of the sensor variants. The dots indicate the mean of the normalized V_o_ (n = 9, 1 pc sensor for each variant), which was measured as a function of the angle (θ) between the excitation current (I = 50 mA) and magnetization (H = 10 Oe). Paired demagnetized (H = 0 Oe) measurements were made for each magnetized measurement.

Nine sensor variants were fabricated with varying sensing areas (i.e., 0.41, 1.15, and 2.75 mm^2^) and film thickness (i.e., 60, 120, 200 nm). Figure 3a shows the fabricated sensor array and the sensor’s microscopic image. The ideal sensor was expected to exhibit a single magnetic domain behaviour with uniform magnetization, where the highest magnitude would be at π/4 and V_o_ would be 0 after demagnetization. Figure 3b shows the effective single-domain behaviour of the fabricated sensors, where V_o_ was measured as a function of the angle (θ) between the excitation current (I = 50 mA) and magnetization (H = 10 Oe). The angle was changed using the senor holder with a step size of 9°. The sensor reached maximum magnitude when the sensor was magnetized at π/4 to the excitation current based on the Equation (1), where the hall voltage varied by sin2θ. The signal dropped to zero while the magnetic field was removed or demagnetized. In Figure 3b, ~9° shift of the magnetization was due to the experimental setup causing misalignment between the sensor and the Helmholtz coil. The V_o_ variation during demagnetization was due to the sensor’s residual magnetization.

### 3.2. Sensitivity and Dynamic Range

**Figure 4:**
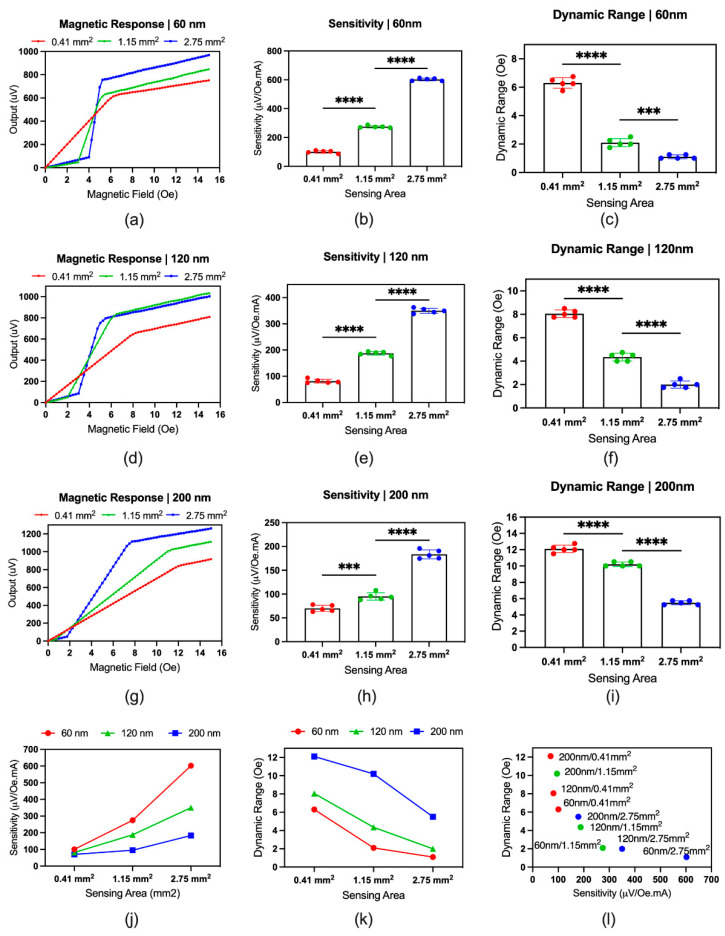
Comparison of the sensor variants: (**a**–**i**) magnetic response, sensitivity, and dynamic range of 60 nm, 120 nm, and 200 nm sensors. In figure (**a**,**d**,**g**) the dots represent the mean of 5 sensors. In figure (**b**,**c**,**e**,**f**,**h**,**i**), the dots represent the individual measurement of different sensors. The statistical significance was based on the ordinary one-way ANOVA (multiple comparison), where *p* = 0.1234 (non-significant, ns), 0.0002 (***), and <0.0001(****). (**j**) Sensitivity comparison of the sensor variants; (**k**) dynamic range comparison of the sensor variants; (**l**) trade-off between sensitivity and dynamic range. In (**j**–**l**), the dot indicates the mean, where *n* = 5.

The sensor output is directly proportional to the excitation current. The sensor dissipates excessive heat with a higher excitation current due to the joule heating, and the output signal increases with high temperatures. Stabilization of the sensor’s temperature is critical for precise measurement. The excitation current should be high enough to mitigate the electrical noise and equivalent magnetic noise. To compare the sensitivity and dynamic range of the sensor variants, the excitation current was maintained to 100 mA, where the sensor’s temperature was 22 °C ± 1 °C. Figure 4a–i depict the sensor variant’s magnetic response, sensitivity, and dynamic range based on thickness. Figure 4j depicts the comparisons based on the sensitivity, where the sensitivity increased with increasing sensing area and decreasing thickness. Figure 4k depicts the comparisons based on the dynamic range, where the dynamic range decreased with increasing sensing area and decreasing thickness.

Combining these results, Figure 4l depicts the trade-off between sensitivity and dynamic range for the sensor variants, where the sensitivity increased with decreasing dynamic range. The results show that the 60 nm/2.75 mm^2^ sensor exhibited the highest sensitivity with the lowest dynamic range.

### 3.3. Magnetic Beads Quantification

**Figure 5:**
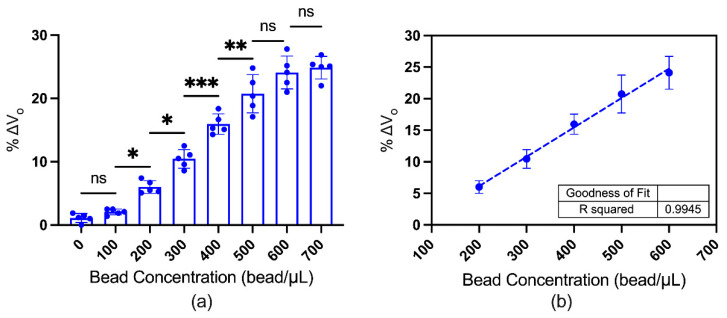
Magnetic beads quantification with the developed EPHE sensor. (**a**) Sensor measurements of the magnetic beads’ droplet with different concentrations. The column indicates the mean, and the error bar indicates the standard deviation, where *n* = 5 per group. Each dot represents the individual measurement of different sensors. The statistical significance was based on the ordinary one-way ANOVA (multiple comparison), where *p* = 0.1234 (non-significant, ns), 0.0332 (*), 0.0021 (**), and <0.0002 (***). (**b**) The linear relationship between sensor output and bead concentration, where the dot with error bar indicates the mean ± SD and the coefficient of determination (R^2^) is 0.9945.

An EPHE sensor with higher sensitivity and lower dynamic range is preferred to quantitate magnetic beads in the biological sample. Based on the findings in Section 3.2, the sensor with 60-nm thick Ni_80_Fe_20_ and 2.75 mm^2^ sensing area was utilized to quantitate the magnetic beads in water. The sensor measurement was performed as described in Section 2.3. Figure 5a shows the sensor measurement of the magnetic beads with concentrations between 0–700 beads/μL in water, with a limit of detection of 200 beads/μL. Figure 5b depicts the sensor’s linear range (200–700 beads/μL) to quantitate the magnetic beads, with a goodness of fit of 0.9945 R^2^. Higher measurement variability was observed in the higher bead concentrations due to the higher aggregation.

## 4. Discussion

In this study, the EPHE sensor was tailored for the magneto-immunodiagnostics application. The sensor’s physical dimension was optimized to quantitate magnetic beads. Nine variants of sensors based on the sensing area (i.e., 0.41 mm^2^, 1.15 mm^2^, and 2.75 mm^2^) and the thin film’s thickness (i.e., 60 nm, 120 nm, 200 nm) were successfully fabricated for optimization. A 3-axis Helmholtz coil and lock-in amplifier-based experimental setup was utilized. The fabricated sensors demonstrated single-domain behaviour by showing that if an external field rotates the magnetization, it returns to the easy axis when the applied magnetic field is removed. Joule heating affects the sensor’s output signal, which increases with the reducing film’s thickness. The output signal increases with the rising temperature. The results show that the sensitivity increased, and the dynamic range decreased with increasing sensing area and decreasing film thickness, and the sensor with 60 nm thickness and 2.75 mm^2^ sensing area had the highest sensitivity and lowest dynamic range. This sensor geometry was tested to quantitate magnetic beads. Sensor measurement was performed with multiple concentrations of the magnetic bead diluted in water. The results indicated the linearity (R^2^ = 0.9945) between 200–600 beads/μL with a limit of detection (LOD) of 200 beads/μL.

Magnetic detection is an emerging technology for the magneto-immunoassay-based POCT device, an alternative to the existing immunodiagnostics. The advantages of magnetic detection are low biological noise, absence of magnetic background signal in the biological samples, low operating power, high scalability, and high sensitivity. A desktop equipment based on magnetic induction for magnetic nanoparticle quantification in the lateral flow test platform is commercially available [42], but is unsuitable for POC application owing to its large size. The application of a magnetic-detection-based POC device is still in infancy. However, several research groups have explored the biomedical application of magnetic detection. Rizzi et al. [43] designed a PHE sensor to detect DNA binding and thermal denaturation. Another study detected the cell-free DNA fragments for cancer diagnostics using an array of 30 GMRs [44]. TMR sensors were reported to detect iron oxide nanoparticle-based magnetic tracers in the complex lymphatic environment [45]. TMR sensor-based microfluidic portable system was reported to detect pathogenic DNA with a sensitivity below the nM range [46]. Lei et al. [47] developed a contactless sensing system to quantitate magnetic nanoparticles bounded on the lateral flow test platform. Amine et al. [48] developed a planar spiral coil-based superparamagnetic beads detection device with a far-reaching aim for magneto-immunoassay application. Although these methods demonstrated high sensitivity, they are limited by expensive fabrication, integration complexity, measurement mechanism, and portability. Hence, the development of a magnetic sensor is required to overcome these limitations.

Previous studies have reported sensor designs using the planar hall effect. One of the simplest designs is the thin film of anisotropic magnetoresistive material (e.g., NiFe, CoFe) deposited on a substrate [40,49]. NiFe sensors exhibited a hall resistance change of 44% with a sensitivity of 900 Ω/T [40]. The main limitation is the insufficient decoupling between the planar hall resistance and the ordinary resistance, which leads to substantial quiescent voltages at the operating point [50]. Numerous studies on the sensing material were carried out using a micro-scaled hall bar to overcome this limitation. Their objective was to probe the magnetization reversal mechanism of the multilayers [51,52,53,54,55]. Substantial effort was placed on the exchange biased hall bar (e.g., NiFe-IrMn system) to achieve linearity between hall voltage and applied magnetic field [56,57,58,59,60]. As a result, the exchange biased hall sensor’s sensitivity was 38–330 Ω/T [29,58,59,61,62,63] with a detection limit of ~10 nT [57]. The advanced hall bridge geometries can further increase the sensitivity, which requires a relatively complex measurement setup [24,25,27,64]. The motive was to develop a sensor appropriate for low-level magnetic field detection. However, the physically large experimental setup is less suitable for the magneto-immunodiagnostic-based point-of-care application. In contrast, the EPHE sensors are more straightforward in design and cheaper than other hall sensors, because the anisotropy is tailored by shape.

In 2010, the pioneering work on the EPHE sensor was conducted by Genish et al. [35], where the numerical simulation and analytical analysis on the shape were conducted. Subsequently, Mor et al. [36] demonstrated the effective single-domain behaviour of the EPHE sensor with a sensitivity of 200 Ω/T. Grosz et al. [22] reported an EPHE sensor design with an equivalent magnetic noise (EMN) of 570 pT/√Hz at optimum excitation current. Later, the EMN was improved to ~200 pT/√Hz using a customized transformer-matched amplifier [21]. More recently, Nhalil et al. [37] incorporated a magnetic flux concentrator (MFC) to increase the EMN to ~5 pT/√Hz. However, the addition of MFC in the EPHE sensor would increase the overall cost and size of any POC device. Moreover, increasing sensitivity using MFCs decreases the dynamic range [65,66]. Hence, MFC should be added only when the sensor’s spatial resolution is insufficient for a particular application. Nhalil et al. [38] reported that the film’s thickness dependency for the EPHE sensor ranged between 25 and 200 nm. The best EMN was ~24 pT/√Hz at 50 nm thickness, but the effect on the dynamic range was not explored. These reports suggest the potential of EPHE sensors for low-level magnetic field detection, and future development focus needs to be directed towards their application.

In the future, the thin film deposition conditions can be further optimized to improve the single-domain behaviour and measurement variation. Growth-induced anisotropy can be incorporated along with shape-induced anisotropy to achieve consistent intrinsic anisotropy. The sensor can be further developed to reduce the noise by optimizing the sensor’s geometrical dimension of the elliptical feature and contact pad junction. The sensitivity can be further improved by incorporating a cooling system, which allows increasing the excitation current. The limit of sensitivity can be improved by further increasing the sensing area. Calibration at different temperatures for magneto-immunoassay application should be performed because the output signal increases with higher temperature. The nano-sized magnetic particles may have a greater advantage than micro-sized particles in achieving faster diffusion, higher surface area to volume ratio, higher binding rates with detection substance, and reduced gaps between the sensor surface and beads. Hence, nano-sized particles may have greater potential for magneto-immunodiagnostics with reduced assay time, increased detection sensitivity, and lower detection limit [67]. The published articles to detect micro/nanomagnetic particles [68,69,70] are incompatible for comparison with the developed senor in this study. Instead, the efficiency comparison would be more appropriate in terms of the sensitivity, dynamic range, and LOD for biomarker detection, which would be conducted in the future.

## 5. Conclusions

Magnetic beads quantification is an emerging technology for application in magneto-immunoassay. While tremendous resources are available on magnetic field sensing technology for different applications, tailoring the design for medical application is still in infancy. Recently, the shaped tailored magnetic anisotropy-based EPHE sensor was reported, which has high sensitivity for magnetic field detection, but has not been explored for medical application. In this study, the EPHE sensor was developed via dimensional optimization to quantitate magnetic beads. The results indicate that the sensitivity increases and dynamic range decreases with the increasing sensing area and thinner magnetic film. The sensor was tested with water-diluted 4.5 μm magnetic dynabeads, where the linear range was 200–700 bead/μL, and the limit of detection was 200 bead/μL. This study has successfully demonstrated the magnetic bead quantification ability using the EPHE sensor. In the future, a biofunctionalization process can be developed, incorporating magnetic beads and the EPHE sensor for point-of-care diagnostics.

## Figures and Tables

**Table 1:** Dimensions of the sensor variants based on the sensing area.

Parameters	Dimension		
	Variant 1	Variant 2	Variant 3
Long axis (a)	3 mm	5 mm	7.5 mm
Sensing region’s length (d)	1.2 mm	2 mm	3 mm
Short axis (b)	0.375 mm	0.625 mm	0.9375 mm
Width of the voltage measuring junction (e)	0.06 mm	0.10 mm	0.15 mm
Ni_80_Fe_20_ thickness (t)	60, 120, 200 nm		
Axis ratio (a/b)	8		
Sensing area	0.41 mm^2^	1.15 mm^2^	2.75 mm^2^

**Figure A1:**
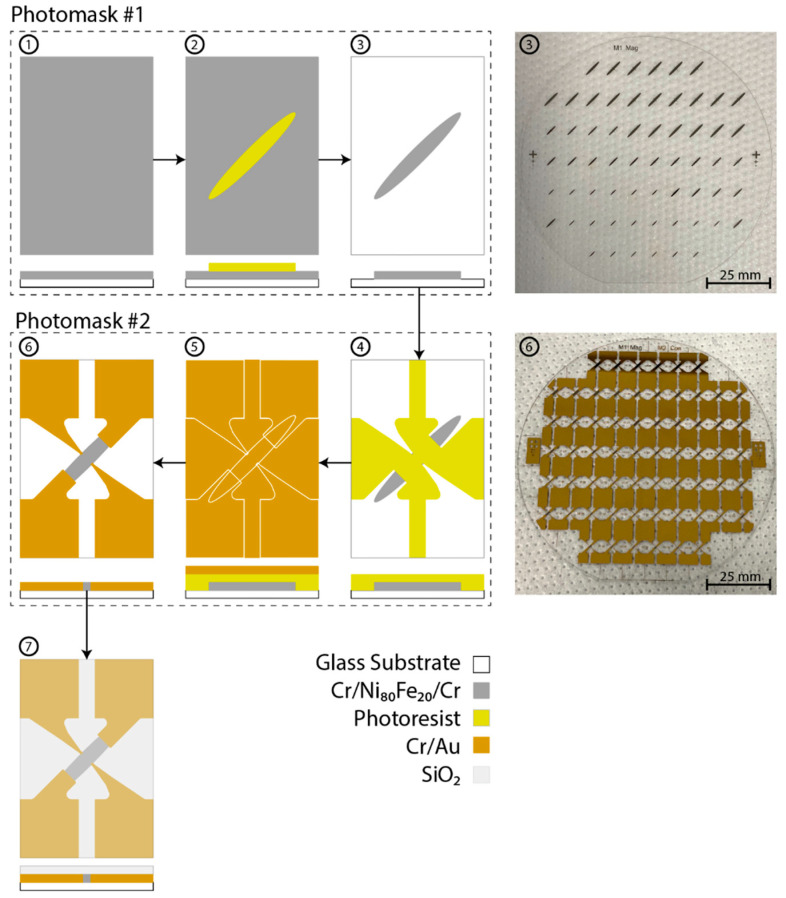
Microfabrication process of the EPHE sensor demonstrating a single sensor’s top and side view. (**1**) EBPVD deposited Cr/Ni_80_Fe_20_/Cr. (**2**) Elliptical feature patterned with photoresist using photomask #1. (**3**) Revealed elliptical thin film after wet-etching, and photo of the actual wafer. (**4**) Contact pad patterned with photoresist using photomask #2. (**5**) EBPVD deposited Cr/Au. (**6**) Revealed contact pad’s thin film after lift-off, and photo of the actual wafer. (**7**) EBPVD deposited SiO_2_ film.

**Figure A2:**
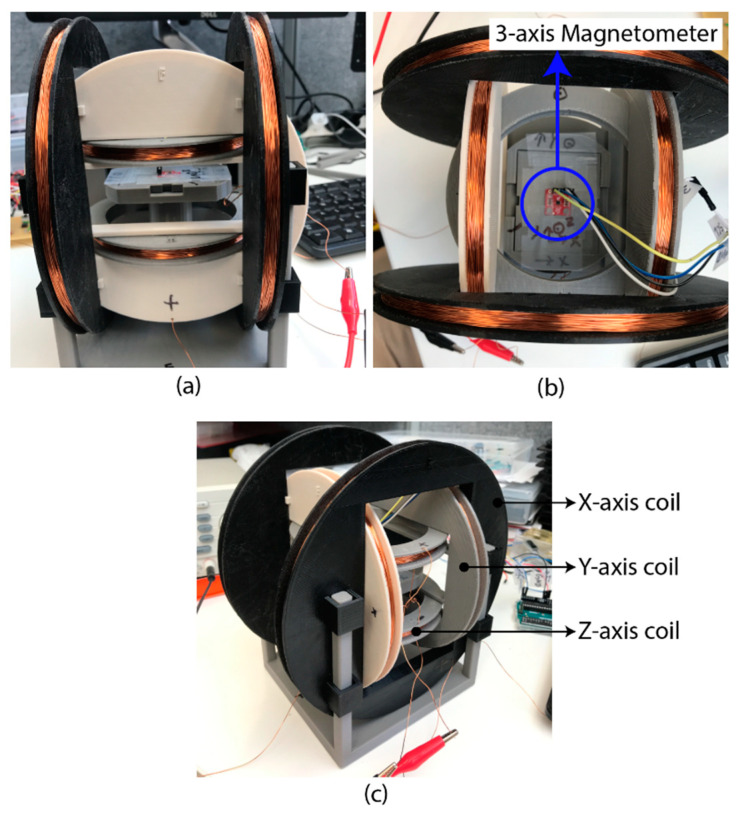
3-aixs Helmholtz magnetic coil. (**a**) side view; (**b**) top view indicating the 3-axis magnetometer mounted for calibration; (**b**) front view; (**c**) isometric view.

**Figure A3:**
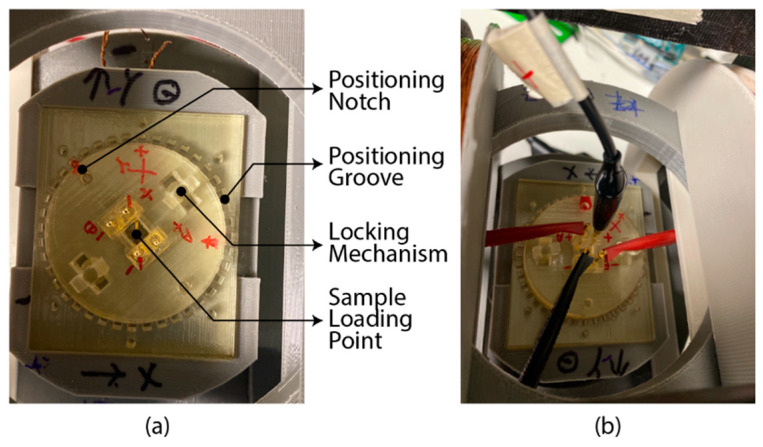
Sensor holder mounted on the 3-axis Helmholtz coil setup for measurement. (**a**) Photograph indicating the sample loading point. (**b**) Electrical connection to the measurement setup.

**Figure A4:**
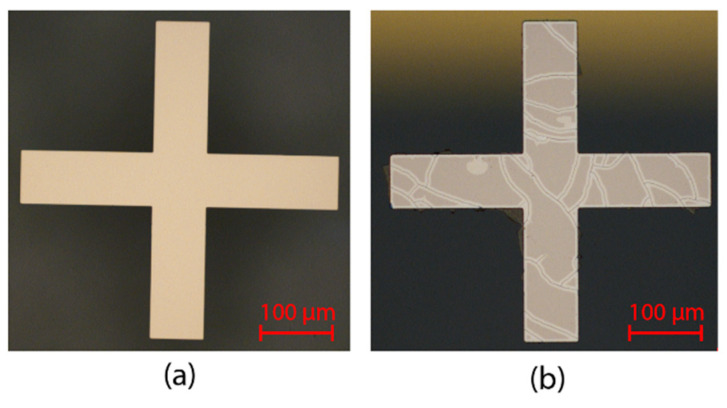
Microscopic image of the photomask alignment mark to indicate the thin film formation quality (**a**) Thin film representing Cr (5 nm)/Ni_80_Fe_20_ (60 nm, 120 nm, and 200 nm)/Cr (5 nm). (**b**) Thin film representing Cr (5 nm)/Ni_80_Fe_20_ (300 nm)/Cr (5 nm).

## Data Availability

Not applicable.

## Appendix A

### Appendix A.1. Microfabrication

The sensor’s microfabrication process had three main steps: (a) patterning the elliptical feature using photomask #1, (b) patterning the contact pad using photomask #2, and (c) SiO_2_ deposition (Figure A1).

1. On polished glass substrate (thickness 0.5 mm), Cr (5 nm)/Ni80Fe20 (60 nm, 120 nm, and 200 nm)/Cr (5 nm) was deposited using electron-beam physical vapor deposition (EBPVD) technique (Equipment: Intlvac Nanochrome II, Intlvac Inc, Georgetown, Canada) at 4 × 10^−6^–8 × 10^−6^ T chamber pressure with a rate of 0.3 Å/s, 2.0 Å/s, and 0.3 Å/s, respectively.
2. The elliptical feature was patterned with the 3.5 µm thick negative photoresist (product id: AZ nLOF 2035, Microchemicals GmbH, Nicolaus-Otto-Straße 39, Germany) using the photolithography process. The process involves mainly two equipment, i.e., mask aligner (model: EVG 620, EV Group, DI-Erich-Thallner-Straße 1, Austria) and spin coater (model: Suss Delta 80). The process included hexamethyldisilazane (HDMS) (manufacturer: Microchemicals GmbH, Nicolaus-Otto-Straße 39, Germany) coating at 3000 rpm followed by immediate softbake at 110 °C for 90 s, photoresist coating at 3000 rpm followed by immediate softbake at 110 °C for 120 s, UV exposure at 100 mJ/cm^2^ dose with the photomask #1, post-exposure bake at 110 °C for 90 s, and pattern development with the developer solution (product id: AZ726, Microchemicals GmbH, Nicolaus-Otto-Straße 39, Germany) for 45 s. The photomasks were produced by the ‘Melbourne Centre for Nanofabrication’.
3. The wet etching process was conducted in two steps. First, the wafer was etched with 33% hydrochloric acid at 50 °C for 10–20 s until the film’s unwanted section turns into black colour. Second, the wafer was etched with chromium etchant at 21 °C for 10–20 s to slow down the process and reveal the elliptical feature.
4. The contact pad feature was patterned with the 1.5 µm thick positive photoresist (product id: AZ1512HS, Microchemicals GmbH, Nicolaus-Otto-Straße 39, Germany) using the photolithography process. The process included HDMS coating at 3000 rpm followed by immediate softbake at 110 °C for 90 s, photoresist coating at 3000 rpm followed by immediate softbake at 110 °C for 90 s, UV exposure at 90 mJ/cm^2^ dose with the photomask #2, and pattern development with the developer solution (product id: AZ726, Microchemicals GmbH, Nicolaus-Otto-Straße 39, Germany) diluted with water (3 developer: 2 water) for 60 s.
5. Cr (10 nm)/Au (300 nm) was deposited using the EBPVD deposition technique at 4 × 10^−6^–8 × 10^−6^ T chamber pressure with a rate of 0.3 Å/s and 2.0 Å/s, respectively.
6. The lift-off process was conducted by ultrasonic cleaning in acetone two times, followed by IPA wash.
7. SiO_2_ (25 nm) was deposited using the EBPVD deposition technique at 4 × 10^−6^–8 × 10^−6^ T chamber pressure with a rate of 0.1 Å/s.
8. The wafer was coated with the AZ1512HS photoresist at 3000 rpm before dicing into single sensors.

### Appendix A.2. Helmholtz Coil

The Helmholtz coil consists of three pairs of coils, where each pair has two identical circular coils placed symmetrically on each side of the sensor along the common axis. The distance between two coils of a pair is equal to the coil’s radius for uniform magnetization. Each coil of a pair is connected in series to a DC power supply. Figure A2 shows photographs of the Helmholtz coil used in this study. The coil was manually wound (100 turns) with enamelled copper wire (diameter 0.5 mm). The inner diameters of the three coils were 180 mm (X-axis), 140 mm (Y-axis), and 100 mm (Z-axis). The magnetic field was controlled by the current, calibrated using a digital 3-axis magnetometer (part no. MAG3110, SparkFun Electronics, Boulder, CO, USA). Figure A2b shows the calibration setup. The magnetic field intensity was directly proportional to the current flowing through the coil. The X-axis, Y-axis, and Z-axis coils generated 59 Oe, 48 Oe, and 78 Oe magnetic field at 5A current, respectively.

### Appendix A.3. Sensor Holder

The sensor holder (Figure A3) mechanism allowed the rotating sensor relative to the magnetic excitation’s direction with a step size of 9°, while the electrical connection was stabilized.

### Appendix A.4. Limitation of the Permalloy Thickness

Four thickness variants of the elliptical pattern were fabricated for optimization. The 60 nm, 120 nm, and 200 nm Ni_80_Fe_20_ patterned was successfully fabricated; Figure A4a represents the variants. The 300 nm Ni_80_Fe_20_ pattern cracked due to thermal expansion (Figure A4b).

## References

[1] Tamanaha C.R., Mulvaney S.P., Rife J.C. Evolution of a magnetic-based biomolecular detection system. Proceedings of the 2009 Annual International Conference of the IEEE Engineering in Medicine and Biology Society.

[2] Tamanaha C.R., Mulvaney S.P., Rife J.C., Whitman L.J. (2008). Magnetic labeling, detection, and system integration. Biosens. Bioelectron..

[3] Tamayo J., Kosaka P.M., Ruz J.J., San Paulo Á., Calleja M. (2013). Biosensors based on nanomechanical systems. Chem. Soc. Rev..

[4] Lai L.M., Goon I.Y., Chuah K., Lim M., Braet F., Amal R., Gooding J.J. (2012). The biochemiresistor: An ultrasensitive biosensor for small organic molecules. Angew. Chem. Int. Ed..

[5] Xianyu Y., Wang Q., Chen Y. (2018). Magnetic particles-enabled biosensors for point-of-care testing. TrAC Trends Anal. Chem..

[6] Chen Y.-T., Kolhatkar A.G., Zenasni O., Xu S., Lee T.R. (2017). Biosensing using magnetic particle detection techniques. Sensors.

[7] Sayad A., Skafidas E., Kwan P. (2020). Magneto-Impedance Biosensor Sensitivity: Effect and Enhancement. Sensors.

[8] Rife J., Miller M., Sheehan P., Tamanaha C., Tondra M., Whitman L. (2003). Design and performance of GMR sensors for the detection of magnetic microbeads in biosensors. Sens. Actuators A Phys..

[9] Cardoso S., Leitao D., Dias T., Valadeiro J., Silva M., Chicharo A., Silverio V., Gaspar J., Freitas P. (2017). Challenges and trends in magnetic sensor integration with microfluidics for biomedical applications. J. Phys. D Appl. Phys..

[10] Lin G., Makarov D., Schmidt O.G. (2017). Magnetic sensing platform technologies for biomedical applications. Lab A Chip.

[11] Zheng C., Zhu K., De Freitas S.C., Chang J.-Y., Davies J.E., Eames P., Freitas P.P., Kazakova O., Kim C., Leung C.-W. (2019). Magnetoresistive sensor development roadmap (non-recording applications). IEEE Trans. Magn..

[12] Baselt D.R., Lee G.U., Natesan M., Metzger S.W., Sheehan P.E., Colton R.J. (1998). A biosensor based on magnetoresistance technology. Biosens. Bioelectron..

[13] Xu L., Yu H., Akhras M.S., Han S.-J., Osterfeld S., White R.L., Pourmand N., Wang S.X. (2008). Giant magnetoresistive biochip for DNA detection and HPV genotyping. Biosens. Bioelectron..

[14] Klein T., Wang W., Yu L., Wu K., Boylan K.L., Vogel R.I., Skubitz A.P., Wang J.-P. (2019). Development of a multiplexed giant magnetoresistive biosensor array prototype to quantify ovarian cancer biomarkers. Biosens. Bioelectron..

[15] Wang T., Zhou Y., Lei C., Luo J., Xie S., Pu H. (2017). Magnetic impedance biosensor: A review. Biosens. Bioelectron..

[16] Sayad A., Uddin S.M., Chan J., Skafidas E., Kwan P. (2021). Meander Thin-Film Biosensor Fabrication to Investigate the Influence of Structural Parameters on the Magneto-Impedance Effect. Sensors.

[17] Mor V., Grosz A., Klein L. (2017). Planar Hall effect (PHE) magnetometers. High Sensitivity Magnetometers.

[18] Thomson W. (1857). XIX. On the electro-dynamic qualities of metals:—Effects of magnetization on the electric conductivity of nickel and of iron. Proc. R. Soc. Lond..

[19] Hung T.Q., Terki F., Kamara S., Kim K., Charar S., Kim C. (2015). Planar Hall ring sensor for ultra-low magnetic moment sensing. J. Appl. Phys..

[20] Freitas P., Ferreira H., Graham D., Clarke L., Amaral M., Martins V., Fonseca L., Cabral J. (2004). Magnetoresistive DNA chips. Magnetoelectronics.

[21] Grosz A., Mor V., Amrusi S., Faivinov I., Paperno E., Klein L. (2016). A high-resolution planar Hall effect magnetometer for ultra-low frequencies. IEEE Sens. J..

[22] Grosz A., Mor V., Paperno E., Amrusi S., Faivinov I., Schultz M., Klein L. (2013). Planar hall effect sensors with subnanotesla resolution. IEEE Magn. Lett..

[23] Elzwawy A., Talantsev A., Kim C. (2018). Free and forced Barkhausen noises in magnetic thin film based cross-junctions. J. Magn. Magn. Mater..

[24] Henriksen A., Dalslet B.T., Skieller D., Lee K., Okkels F., Hansen M.F. (2010). Planar Hall effect bridge magnetic field sensors. Appl. Phys. Lett..

[25] Persson A., Bejhed R.S., Østerberg F.W., Gunnarsson K., Nguyen H., Rizzi G., Hansen M.F., Svedlindh P. (2013). Modelling and design of planar Hall effect bridge sensors for low-frequency applications. Sens. Actuators A Phys..

[26] Østerberg F.W., Rizzi G., Hansen M.F. (2013). On-chip measurements of Brownian relaxation of magnetic beads with diameters from 10 nm to 250 nm. J. Appl. Phys..

[27] Persson A., Bejhed R.S., Nguyen H., Gunnarsson K., Dalslet B.T., Oesterberg F.W., Hansen M.F., Svedlindh P. (2011). Low-frequency noise in planar Hall effect bridge sensors. Sens. Actuators A Phys..

[28] Oh S., Le T.T., Kim G., Kim C. (2007). Size effect on NiFe/Cu/NiFe/IrMn spin-valve structure for an array of PHR sensor element. Phys. Status Solidi (A).

[29] Thanh N., Kim K., Kim C., Shin K., Kim C. (2007). Microbeads detection using Planar Hall effect in spin-valve structure. J. Magn. Magn. Mater..

[30] Oh S., Baek N.S., Jung S.-D., Chung M., Hung T.Q., Anandakumar S., Sudha Rani V., Jeong J.-R., Kim C. (2011). Selective binding and detection of magnetic labels using PHR sensor via photoresist micro-wells. J. Nanosci. Nanotechnol..

[31] Østerberg F.W., Rizzi G., de la Torre T.Z.G., Strömberg M., Strømme M., Svedlindh P., Hansen M. (2013). Measurements of Brownian relaxation of magnetic nanobeads using planar Hall effect bridge sensors. Biosens. Bioelectron..

[32] Qejvanaj F., Zubair M., Persson A., Mohseni S., Fallahi V., Sani S.R., Chung S., Le T., Magnusson F., Åkerman J. (2014). Thick double-biased IrMn/NiFe/IrMn planar hall effect bridge sensors. IEEE Trans. Magn..

[33] Østerberg F.W., Henriksen A.D., Rizzi G., Hansen M.F. (2013). Comment on “Planar Hall resistance ring sensor based on NiFe/Cu/IrMn trilayer structure” [J. Appl. Phys. 113, 063903 (2013)]. J. Appl. Phys..

[34] Sinha B., Quang Hung T., Sri Ramulu T., Oh S., Kim K., Kim D.-Y., Terki F., Kim C. (2013). Planar Hall resistance ring sensor based on NiFe/Cu/IrMn trilayer structure. J. Appl. Phys..

[35] Genish I., Shperber Y., Naftalis N., Salitra G., Aurbach D., Klein L. (2010). The effects of geometry on magnetic response of elliptical PHE sensors. J. Appl. Phys..

[36] Mor V., Schultz M., Sinwani O., Grosz A., Paperno E., Klein L. (2012). Planar Hall effect sensors with shape-induced effective single domain behavior. J. Appl. Phys..

[37] Nhalil H., Givon T., Das P.T., Hasidim N., Mor V., Schultz M., Amrusi S., Klein L., Grosz A. (2019). Planar Hall effect magnetometer with 5 pT resolution. IEEE Sens. Lett..

[38] Nhalil H., Das P.T., Schultz M., Amrusi S., Grosz A., Klein L. (2020). Thickness dependence of elliptical planar Hall effect magnetometers. Appl. Phys. Letters..

[39] Valadeiro J., Leitao D., Cardoso S., Freitas P. (2017). Improved efficiency of tapered magnetic flux concentrators with double-layer architecture. IEEE Trans. Magn..

[40] Roy A., Kumar P.A. (2010). Giant planar Hall effect in pulsed laser deposited permalloy films. J. Phys. D Appl. Phys..

[41] Marinace J.C. (1961). High Sensitivity Hall Effect Probe. Google Patents.

[42] MagnaBioSciences. http://www.magnabiosciences.com.

[43] Rizzi G., Østerberg F.W., Henriksen A.D., Dufva M., Hansen M.F. (2015). On-chip magnetic bead-based DNA melting curve analysis using a magnetoresistive sensor. J. Magn. Magn. Mater..

[44] Dias T., Cardoso F., Martins S., Martins V., Cardoso S., Gaspar J., Monteiro G., Freitas P. (2016). Implementing a strategy for on-chip detection of cell-free DNA fragments using GMR sensors: A translational application in cancer diagnostics using ALU elements. Anal. Methods.

[45] Cousins A., Balalis G., Thompson S.K., Morales D.F., Mohtar A., Wedding A., Thierry B. (2015). Novel handheld magnetometer probe based on magnetic tunnelling junction sensors for intraoperative sentinel lymph node identification. Sci. Rep..

[46] Sharma P.P., Albisetti E., Massetti M., Scolari M., La Torre C., Monticelli M., Leone M., Damin F., Gervasoni G., Ferrari G. (2017). Integrated platform for detecting pathogenic DNA via magnetic tunneling junction-based biosensors. Sens. Actuators B. Chem..

[47] Lei H., Wang K., Ji X., Cui D. (2016). Contactless measurement of magnetic nanoparticles on lateral flow strips using tunneling magnetoresistance (TMR) sensors in differential configuration. Sensors.

[48] Rabehi A., Garlan B., Achtsnicht S., Krause H.-J., Offenhäusser A., Ngo K., Neveu S., Graff-Dubois S., Kokabi H. (2018). Magnetic detection structure for lab-on-chip applications based on the frequency mixing technique. Sensors.

[49] Jen S., Wang P., Tseng Y., Chiang H.-P. (2009). Planar Hall effect of Permalloy films on Si (111), Si (100), and glass substrates. J. Appl. Phys..

[50] Montaigne F., Schuhl A., Van Dau F.N., Encinas A. (2000). Development of magnetoresistive sensors based on planar Hall effect for applications to microcompass. Sens. Actuators A Phys..

[51] Ko T., Park B., Lee J., Rhie K., Kim M., Rhee J. (1999). Planar Hall effect of glass/Fe70 Å/[Co21 Å/Cu25 Å] 20 multilayers. J. Magn. Magn. Mater..

[52] Adeyeye A., Win M., Tan T., Chong G., Ng V., Low T. (2004). Planar Hall effect and magnetoresistance in Co/Cu multilayer films. Sens. Actuators A Phys..

[53] Chang Y., Chang C., Wu J.-C., Wei Z., Lai M., Chang C. (2006). Probing the magnetization reversal of microstructured permalloy cross by planar hall measurement and magnetic force microscopy. IEEE Trans. Magn..

[54] Jen S., Lee J., Yao Y., Chen W. (2001). Transverse field dependence of the planar Hall effect sensitivity in Permalloy films. J. Appl. Phys..

[55] Morvic M., Betko J. (2005). Planar Hall effect in Hall sensors made from InP/InGaAs heterostructure. Sens. Actuators A Phys..

[56] Schuhl A., Van Dau F.N., Childress J. (1995). Low-field magnetic sensors based on the planar Hall effect. Appl. Phys. Lett..

[57] Van Dau F.N., Schuhl A., Childress J., Sussiau M. (1996). Magnetic sensors for nanotesla detection using planar Hall effect. Sens. Actuators A Phys..

[58] Ejsing L., Hansen M.F., Menon A.K., Ferreira H., Graham D., Freitas P. (2004). Planar Hall effect sensor for magnetic micro-and nanobead detection. Appl. Phys. Lett..

[59] Ejsing L., Hansen M.F., Menon A.K., Ferreira H.A., Graham D.L., Freitas P.P. (2005). Magnetic microbead detection using the planar Hall effect. J. Magn. Magn. Mater..

[60] Tu B.D., Danh T.M., Duc N.H. (2009). Optimization of planar Hall effect sensor for magnetic bead detection using spin-valve NiFe/Cu/NiFe/IrMn structures. Proc. J. Phys. Conf. Ser..

[61] Hung T.Q., Oh S., Jeong J.-R., Kim C. (2010). Spin-valve planar Hall sensor for single bead detection. Sens. Actuators A Phys..

[62] Damsgaard C.D., Freitas S.C., Freitas P.P., Hansen M.F. (2008). Exchange-biased planar Hall effect sensor optimized for biosensor applications. J. Appl. Phys..

[63] Hung T.Q., Oh S., Sinha B., Jeong J.-R., Kim D.-Y., Kim C. (2010). High field-sensitivity planar Hall sensor based on NiFe/Cu/IrMn trilayer structure. J. Appl. Phys..

[64] Pişkin H., Akdoğan N. (2019). Interface-induced enhancement of sensitivity in NiFe/Pt/IrMn-based planar hall sensors with nanoTesla resolution. Sens. Actuators A Phys..

[65] Lee S.-K., Romalis M. (2008). Calculation of magnetic field noise from high-permeability magnetic shields and conducting objects with simple geometry. J. Appl. Phys..

[66] Griffith W.C., Jimenez-Martinez R., Shah V., Knappe S., Kitching J. (2009). Miniature atomic magnetometer integrated with flux concentrators. Appl. Phys. Lett..

[67] Tang C., He Z., Liu H., Xu Y., Huang H., Yang G., Xiao Z., Li S., Liu H., Deng Y. (2020). Application of magnetic nanoparticles in nucleic acid detection. J. Nanobiotechnology.

[68] Ejsing L.W. (2006). Planar Hall Sensor for Influenza Immunoassay.

[69] Sinha B., Anandakumar S., Oh S., Kim C. (2012). Micro-magnetometry for susceptibility measurement of superparamagnetic single bead. Sens. Actuators A Phys..

[70] Hung T.Q., Kim D.Y., Rao B.P., Kim C., Rinken T. (2013). Novel Planar Hall Sensor for Biomedical Diagnosing Lab-on-a-Chip. State of the Art in Biosensors—General Aspects.

